# Obesity and postoperative outcomes of the patients with laparoscopic adrenalectomy: a systematic review and meta-analysis

**DOI:** 10.1186/s12893-020-00848-y

**Published:** 2020-08-31

**Authors:** Celestin Danwang, Valirie Ndip Agbor, Jean Joel Bigna

**Affiliations:** 1 Epidemiology and Biostatistics Unit, Institute of Experimental and Clinical Research, Université Catholique de Louvain, Brussels, Belgium; 2 Department of Surgery and Specialties, Faculty of Medicine and Biomedical Sciences, University of Yaoundé I, Yaoundé, Cameroon; 3 Clinical Trial Service Unit and Epidemiological Studies Unit, Nuffield Department of Population Health, University of Oxford, Oxford, UK; 4 Department of Clinical Research, Health Education and Research Organisation (HERO), Buea, Cameroon; 5 Department of Epidemiology and Public Health, Centre Pasteur of Cameroon, Yaoundé, Cameroon; 6 School of Public Health, Faculty of Medicine, University of Paris Sud XI, Paris, France

**Keywords:** Laparoscopy, Adrenalectomy, Obesity, Outcome

## Abstract

**Background:**

Studies have suggested differences in postoperative outcomes between patients with obesity and those without following adrenalectomy, but these remained to be ascertained with synthesis of available evidence. The aim of this systematic review and meta-analysis was to investigate the association between obesity and outcomes of patients after laparoscopic adrenalectomy.

**Methods:**

We searched EMBASE, PubMed, Global Index Medicus, and Web of Science, without language restriction, to identify cohort studies published between January 1, 2000 and November 6, 2019. We considered studies with data comparing outcomes of adults with and without obesity after laparoscopic adrenalectomy. Random-effects meta-analysis was used to pool study-specific estimates. This review was registered with PROSPERO, CRD42018117070.

**Results:**

Five studies with data on a pooled sample of 353 patients with obesity and 828 without were included in the meta-analysis. The risk of bias was moderate to low. We found no association between obesity and the various stages of postoperative complications: Clavien-Dindo grade 1 (OR = 1.57; 95%CI = 0.55–4.48; I^2^ = 44.6%), grade 2 (OR = 1.12; 95%CI = 0.54–2.32; I^2^ = 0.0%), grade 3 (OR = 1.79; 95%CI = 0.58–5.47; I^2^ = 0.0%;), grade 4 (OR = 0.43; 95%CI = 0.05–3.71; I^2^ = 0.0%), and grade 5 (death) (OR = 0.43; 95% CI = 0.02–14.31). Furthermore, no association was found between obesity and readmission rates (OR = 0.7; 95% CI = 0.13–3.62) and conversion of laparoscopic to open surgery (OR = 0.62; 95% CI = 0.16–2.34; I^2^ = 19.5%).

**Conclusions:**

This study suggests that obesity is not associated with complications following laparoscopic adrenalectomy. This meta-analysis might have been underpowered to detect a true association between obesity and patient outcome after laparoscopic adrenalectomy due to the small number of included studies. Larger studies are needed to clarify the role of obesity in patients undergoing laparoscopic adrenalectomy.

## Background

Obesity is a global public health concern, with rising prevalence over the last forth decade [1–4]. Low- and middle-income countries are experiencing an epidemiologic transition from communicable to chronic non-communicable diseases [3]. Hypertension, diabetes, tobacco, and obesity are among the leading risk factors for non-communicable disease [3, 4]. According to the World Health Organization (WHO), over 1.9 billion adults were overweight in 2016 [2, 5]. Of these over 650 million were obese. Moreover, 39% of adults aged 18 years and over were overweight and 13% were obese during the same year [2, 5]. Thus, obesity represents a global public health and clinical concern face by teams working in both the medical and surgical fields.

The role of obesity in the outcome of patients after surgery in general, and after abdominal and adrenal surgery in particular is unclear [6–12]. Some studies suggested that obesity has a detrimental effect on the outcomes of patients after abdominal surgery [8, 13–15]. It is argued that patients with obesity would have higher postoperative morbidity than patients without [16–18]. On the other hand, other studies evoked the absence of association between obesity and occurrence of post-operative complication, and even a protective effect of obesity on postoperative mortality after digestive surgery, and this phenomenon has been named the ‘obesity paradox’ [7, 19, 20]. Unfortunately, most of these studies either focused on surgery of intraperitoneal organs or a particular type of surgery (cancer surgery or bariatric surgery), leaving aside the surgical procedures of retro-peritoneal structures such as adrenal glands and benign tumors.

Adrenal glands are two retroperitoneal structures located on the kidneys, divided in two part: cortex and medulla [21]. Tumors such as adenomas and adrenocortical carcinomas that need surgical interventions can originate from the adrenal glands [22]. During the last two decades, surgery of adrenal gland has been widely vulgarized, and the number of published studies on this topic has increased significantly. This phenomenon is partly due to the ageing of the global population and improved access to modern medical imaging such as computed tomography and magnetic resonance imaging which allow a more frequent fortuitous discovery of adrenal tumors named incidentalomas [23–27]. These tumors are among the most common indications of adrenalectomies, regardless of the suspected histological nature [28]. Laparoscopic surgery has gradually replaced open surgery as gold standard for the management of adrenal tumors [17, 29–33]. Although indications for this approach were initially limited to benign tumors, they gradually expanded to include malignant tumors, making laparoscopy the preferred approach for adrenal gland surgery [30–32, 34, 35]. However, laparoscopic adrenalectomy is said to be technically difficult in patients with obesity given the retroperitoneal localization of adrenal glands. Furthermore, several recent studies suggested a difference in outcome of patients having laparoscopic adrenalectomy when stratified according to obesity status [17, 20]. Hitherto, no study has ascertained this hypothesis by the mean of a meta-analysis of available published data. With the increased number of adrenal gland surgery worldwide, performed especially in obese patients, there is a need to generate reliable evidence on the effect of obesity on patient outcome following laparoscopic adrenalectomy. Thus, the present systematic review and meta-analysis aimed at assessing the association between obesity and postoperative outcomes of patients after laparoscopic adrenalectomy.

## Methods

This review was registered in the International Prospective Register of Systematic Reviews (PROSPERO) under the registration number CRD42018117070. We used Preferred Reporting Items for Systematic reviews and Meta-Analyses (PRISMA) guidelines as template for reporting this systematic review with meta-analysis [36].

### Search strategy and selection criteria

Global Index Medicus, Excerpta Medica Database (EMBASE), Medline through PubMed, and Web of Science (Web of Science Core Collection, Current Contents Connect, KCI-Korean Journal Database, SciELO Citation Index, Russian Science Citation Index) were searched to identify studies published between January 1, 2000 and November 6, 2019. No language restriction was applied. The initial search strategy was designed for EMBASE and was adapted for use in other databases (Supplementary Table 1). The search strategy was based on the combination of relevant text words and medical subject headings related to adrenalectomy and obesity. Moreover, the references of all relevant articles found were scrutinized for potential additional data sources. For studies published in more than one report, the one reporting the largest sample size was considered.

Our population of interest was patients with adrenalectomy regardless of age, sex, and geographic location. Exposure considered for this review was obesity defined as body mass index (BMI) ≥ 30 Kg/m^2^ or central obesity for waist circumference or waist-to-hip ratio as per WHO guidelines or according to country specific guidelines [5, 37]. Outcomes of interest included overall deaths, surgical site infection, conversion from laparoscopy to open surgery, and complications classified according to Clavien-Dindo grades [38]. We considered prospective and retrospective cohort studies. We excluded letters, reviews, commentaries, editorials, studies lacking key data and/or explicit method description as well as studies in which relevant data on obesity and outcomes of patients after adrenalectomy was not possible to extract even after contacting the corresponding author.

Two reviewers (CD and VNA) independently screened the titles and abstract of articles for eligibility. Full texts of potentially eligible articles were retrieved and screened for final inclusion. Disagreements between the two reviewers were solved by discussion and when a consensus was not reached, a third reviewer (JJB) resolved discrepancies.

### Data synthesis and analysis

A standardized data extraction sheet was used by two reviewers (VNA and CD) to independently extract data from individual studies. The last name of the first author, year of publication, country, study design, sample size, mean or median age, proportion of males, mean/median duration of hospital stay, mean/median BMI, mean/median WC, definition of obesity, total number of obese and non-obese patients included, number of patients with outcomes of interest.

A meta-analysis was used to summarize evidence. Study-specific estimates were pooled through a Dersimonian and Laird random-effects meta-analysis model to obtain an overall summary estimate with inverse variance method [39]. Odds ratio (OR), with their 95% confidence interval (95%CI), was used as the effect size. The presence of heterogeneity was assessed with the χ^2^ test of Cochrane and, quantified by I^2^ values, assuming that I^2^ of 25, 50 and 75% represent low, medium and high heterogeneity respectively [40]. A *p* value < 0.05 was indicative of significant difference. Inter-rater agreement for study inclusion was assessed using Cohen’s κ coefficient [41]. The methodological quality of included study was assessed by two reviewers (VNA and CD) using an adapted version of the risk of bias in non-randomized studies of interventions (Supplementary Table 2) [42].

## Results

### Study selection

In total, 387 citations were identified from database searches. Twenty-four eligible full texts were assessed for eligibility after duplicate citations have been removed and records eliminated following screening of title and abstract. Five studies met the inclusion criteria and were included in this systematic review and meta-analysis [20, 43–46]. Supplementary Figure 1 displays the process of study selection including the reasons of study exclusion of full texts assessed for eligibility. The Cohen’s coefficients for the inclusion of studies based on titles and abstract and full text were 0.91 and 0.94, respectively.

### Characteristics of included studies

Studies included were published between 2011 and 2018 and conducted between 1997 and 2016. Studies were from China, Japan, Norway, Poland, and USA; one study per country. The timing of data collection was retrospective for three studies, prospective for one, and unclear for one study. All studies considered adult participants (≥ 18 years) with the mean/median age varying from 44 to 59 years for patients with obesity and from 49 to 57 years for those without. Male proportion varied from 29.3 to 54.0% in patients with obesity and from 33.1 to 54.8% for those without obesity. The mean duration for surgical procedures varied from 1.5 to 3.8 h for patients with obesity and from 1.2 to 3.5 h for non-obese patients. The mean duration for hospital staying after surgery varied from 2 to 6 days for participants with obesity and from 1 to 6 days for non-obese ones. All studies defined obesity with BMI ≥ 30 mg/m^2^. There were various indications for adrenalectomy (Supplementary Table 3).

For all the five included studies, there was high risk of confounding bias for one study, high risk for selection bias in two studies, high risk of bias due to missing data in one study. All studies had low risk of classification bias and unclear risk of bias in outcome measurement (Supplementary Figure 2).

### Postoperative complications of laparoscopic adrenalectomy

**Fig. 1:**
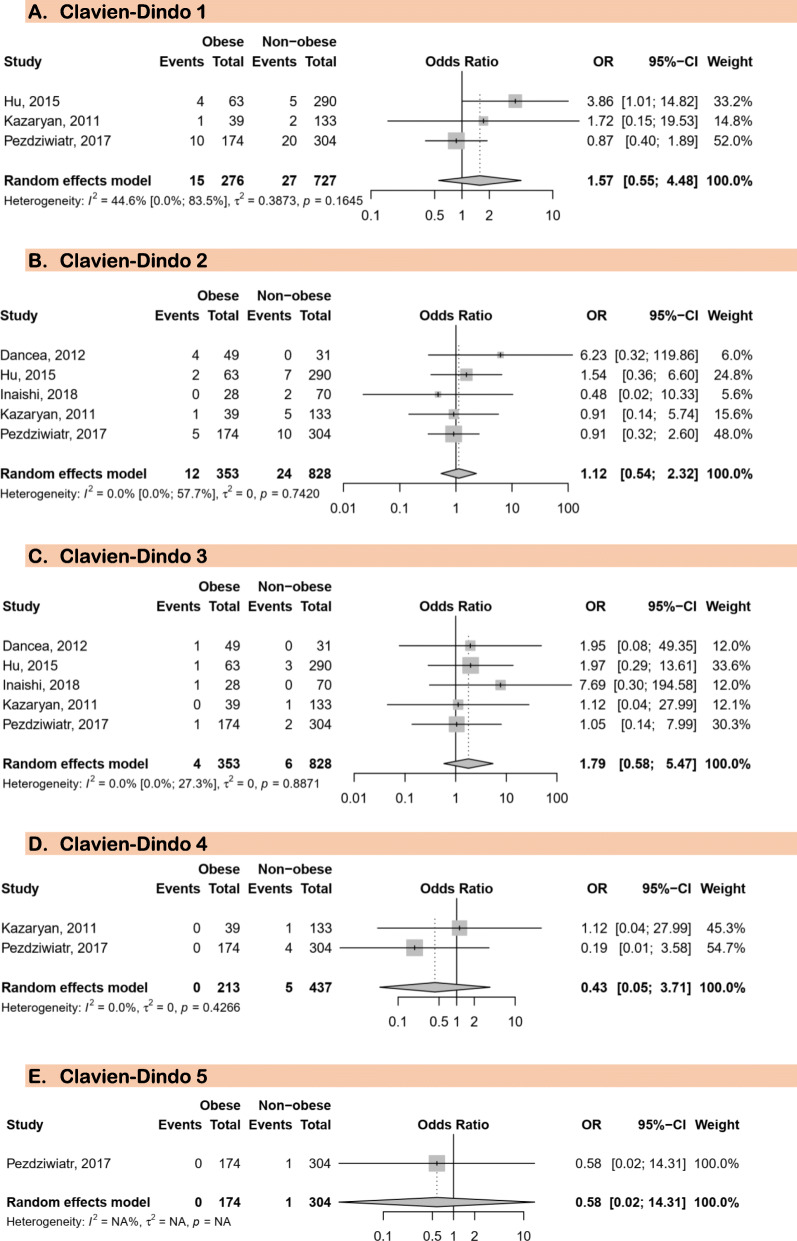
Meta-analysis of postoperative complications after laparoscopic adrenalectomy in patients with and without obesity

In total, five studies were included for this analysis [20, 43–46]. There was no association between obesity and all Clavien-Dindo grades for postoperative complications (Fig. 1): Clavien-Dindo grade 1 (OR 1.57; 95%CI: 0.55–4.48), Clavien-Dindo grade 2 (OR 1.12; 95%CI: 0.54–2.32), Clavien-Dindo garde 3 (OR 1.79; 95%CI: 0.58–5.47), Clavien-Dindo grade 4 (OR 0.43; 95%CI: 0.05–3.71), and Clavien-Dindo grade (death) 5 (OR 0.43; 95%CI: 0.02–14.31). Three out of five study reported no difference in intervention time between patients with and those without obesity. Furthermore, there was no substantial heterogeneity for all these analyses.

### Conversion from laparoscopic to open adrenalectomy

**Fig. 2:**
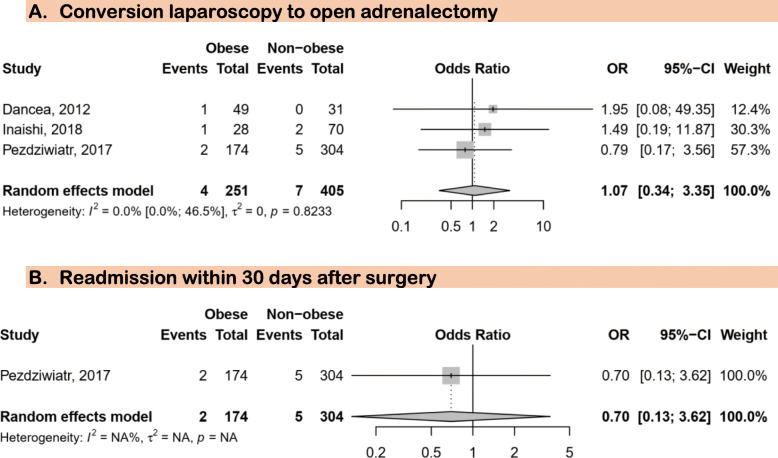
Association between obesity and conversion from laparoscopic to open surgery and readmission within 30 days after surgery

Three studies were eligible for this analysis [43, 45, 46]. There was no association between obesity and conversion of laparoscopy to open adrenalectomy (OR 1.07; 95%CI: 0.34–3.35) (Fig. 2).

### Readmission within 30 days after surgery

One study provided data on readmission as a complication of laparoscopic adrenalectomy [46]. We found no association between obesity and the risk of readmission within 30 days of surgery (OR 0.7; 95%CI: 0.13–3.62) (Fig. 2).

## Discussion

From five cohort studies with 353 patients with obesity and 828 without obesity, we found that obesity was not associated with the occurrence of postoperative complications, risk of death, conversion during laparoscopic adrenalectomy to open surgery and, the readmission within 30 days after surgery. Definitively, evidence suggesting that obesity is a risk factor for increased postoperative morbidity during and after surgery are contradictory [6, 11, 16, 19, 47, 48].

The observed absence of difference in outcome between obese and non-obese patients following laparoscopic adrenalectomy is probably multifactorial and related to the timing of this type of surgery, mechanical constraints and postoperative management [33, 49]. In fact, laparoscopic adrenalectomy is usually performed as a planned surgery. Thus, tailored preoperative preparation can be done to minimize the surgical risk by taking into consideration the particularity of each patient in terms of co-morbidities and obesity [50, 51]. Furthermore, during laparoscopic adrenalectomy, parietal thickness is not really a major factor. Regardless of wall thickness, the incision is minimal and is associated with minimal exposure and manipulation of subcutaneous tissue and muscle, reducing the risk of post-operative infection and pain, regardless of BMI [7, 52]. Moreover, after a laparoscopic adrenalectomy, feeding is quickly resumed and rapid recovery is expected for both obese and non-obese patients [53]. Which reduced significantly the risk of complication related to prolong bed rest and fasting in patients with obesity.

One of the most important elements related to patient outcomes during and after laparoscopic surgery is the visceral fat mass rather than obesity estimated with BMI [54–56]. It is argued that some populations such as Asians may have a BMI in the normal range, but a high visceral fat content that may compromise the per- and postoperative outcomes [55]. Because laparoscopic adrenalectomy is performed within the intracorporeal field, it is more sensitive to element that can modify intra or retroperitoneal environment like visceral fat. This may explain why we observed to evidence of an association between BMI and patient outcome following laparoscopic adrenalectomy when stratified according to obesity status, but a difference may be observe if those patients are stratified instead according to their visceral fat mass [55, 57, 58].

Our findings were similar to those of previous studies which found no difference in post-operative outcomes between patients with and without obesity after digestive tract surgery [10, 48, 59, 60], gynecologic surgery [61, 62], vascular surgery [63, 64], neurosurgery [9], or orthopedic surgery [12, 65, 66], even among those admitted in intensive care unit [67]. In most of these studies, a minimal invasive surgical method was used like in studies included in the current meta-analysis, allowing less damages for surrounding tissues, rapid post-operative rehabilitation,

Elsewhere, an increased risk of morbidity in postoperative period in patients with obesity has been widely documented in recent years in both oncology and non-oncology surgery. In the field of oncological surgery of the digestive tract, recent studies suggested obesity as risk factor of postoperative complications such as anastomosis leakage, surgical site infection, cardiovascular complications, however without an increased risk of mortality [7, 14, 15, 18, 19].

The results of this meta-analysis should be considered in the context of some limitations. The first is the small number of studies included in the meta-analysis. Indeed, only five were eligible for the meta-analysis. Therefore, this study might have been underpowered to detect an association if one truly exists. The high cost related to equipment necessary for laparoscopic surgery could explain the low number of eligible studies done in low-incomes countries; this can hinder the generalizability of the findings. However, this is the first systematic review and meta-analysis to summarized current evidence on the effect of obesity on outcome of patients after laparoscopic surgery. No substantial heterogeneity was found in all analyses.

## Conclusions

This study suggests that obesity is not associated with adverse outcomes following laparoscopic adrenalectomy. However, larger scale studies are needed to ascertain the impact of obesity on postoperative outcomes among patients undergoing adrenalectomy.

## Supplementary information

**Additional file 1.**

## Data Availability

All data generated or analyzed during this study are included in this published article and its supplementary information files.

## Abbreviations

BMI: Body mass index
WHO: World Health Organization
