# Supplementary material for: Obesity and postoperative outcomes of the patients with laparoscopic adrenalectomy: a systematic review and meta-analysis
Source: BMC Surg. 2020 Aug 31;20:194. doi: 10.1186/s12893-020-00848-y (PMC7457363; doi:10.1186/s12893-020-00848-y)
Supplement: Supplementary file 1 — Additional file 1. [file 12893_2020_848_MOESM1_ESM.docx]

Supplementary Table 1. Search strategy for EMBASE

| **Search** | **Search terms** |
| --- | --- |
| #1 | 'laparoscopy'/exp OR 'laparoscopy' OR 'laparoscopic' OR 'laparoscope'/exp OR 'laparoscope' OR 'laparoscopes'/exp OR 'laparoscopes' OR 'laparoscopies' OR 'celioscopy'/exp OR 'celioscopy' OR 'celioscopies' OR 'periteneoscopy' OR 'periteneoscopies' |
| #2 | 'obesity'/exp OR obesity OR obese OR 'overweight'/exp OR overweight |
| #3 | 'adrenalectomy'/exp OR adrenalectomy OR 'adrenal'/exp OR adrenal |
| #4 | #1 AND #2 AND #3 |

From January 1^st^, 2000 to November 30^th^, 2018


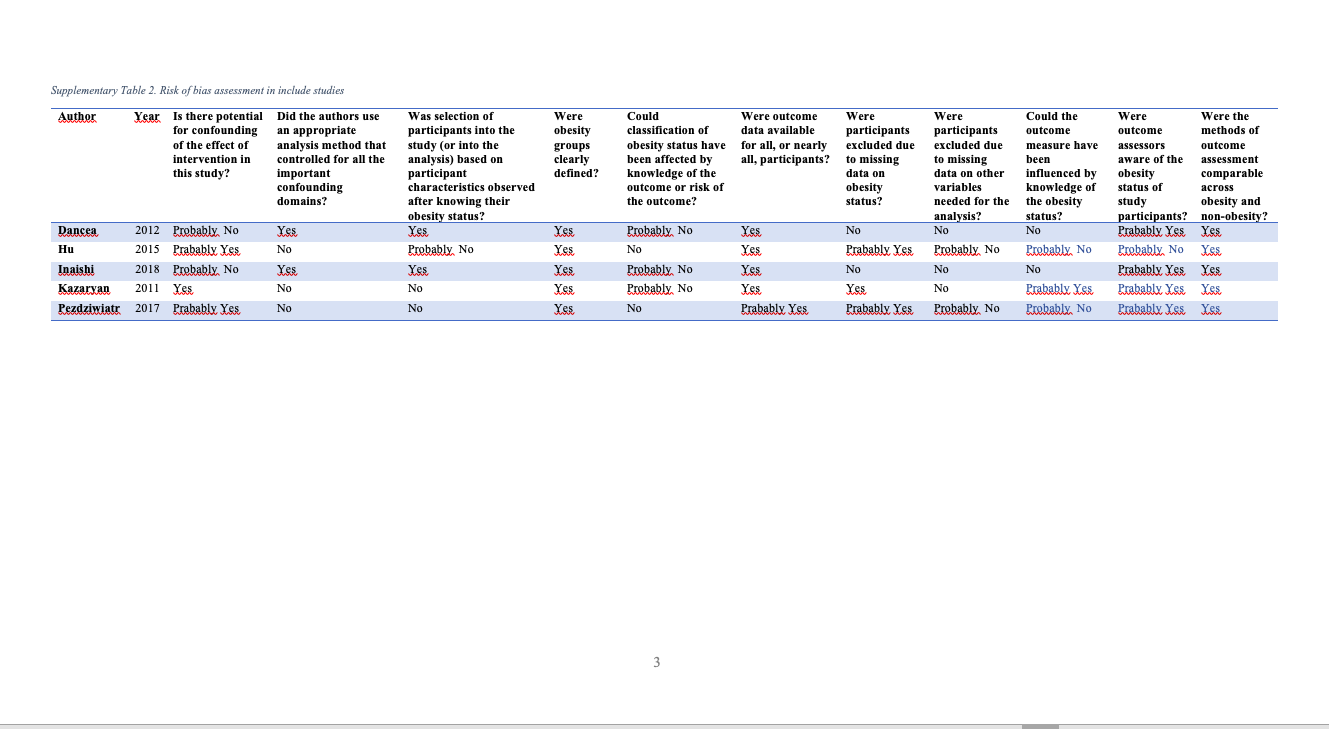


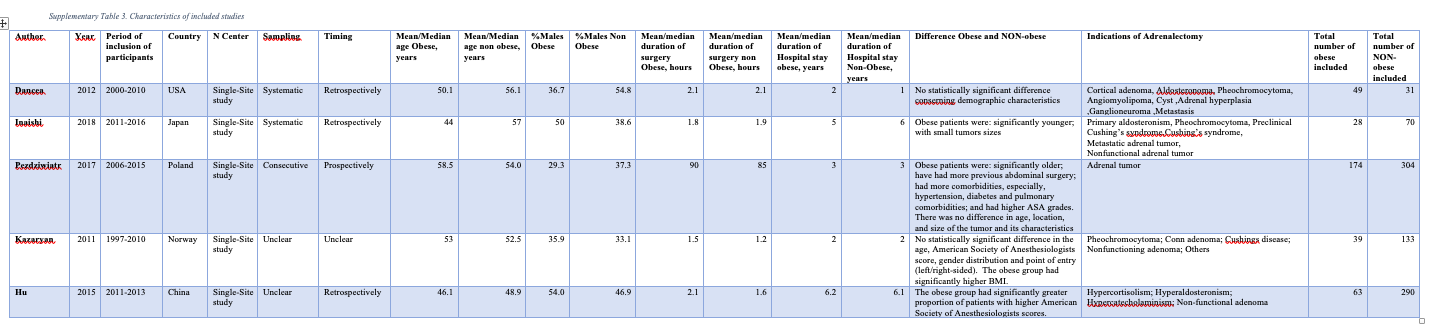


**363 records identified through database searches**

**332 records screened on the basis of title and/or abstract**

**17 full-text articles excluded**

- 12 No obesity population defined
- 3 Not possible to extract key data
- 1 Not possible to differentiate data for renal and adrenal surgery
- 1 No spate data for laparoscopic adrenalectomy

**5 studies included in qualitative synthesis**

**31 duplicates excluded**

**310 records excluded based on title and abstract**

**5 studies included in quantitative synthesis (meta-analysis)**

**22 full-text articles assessed for eligibility**

Supplementary Figure 1. Study selection process


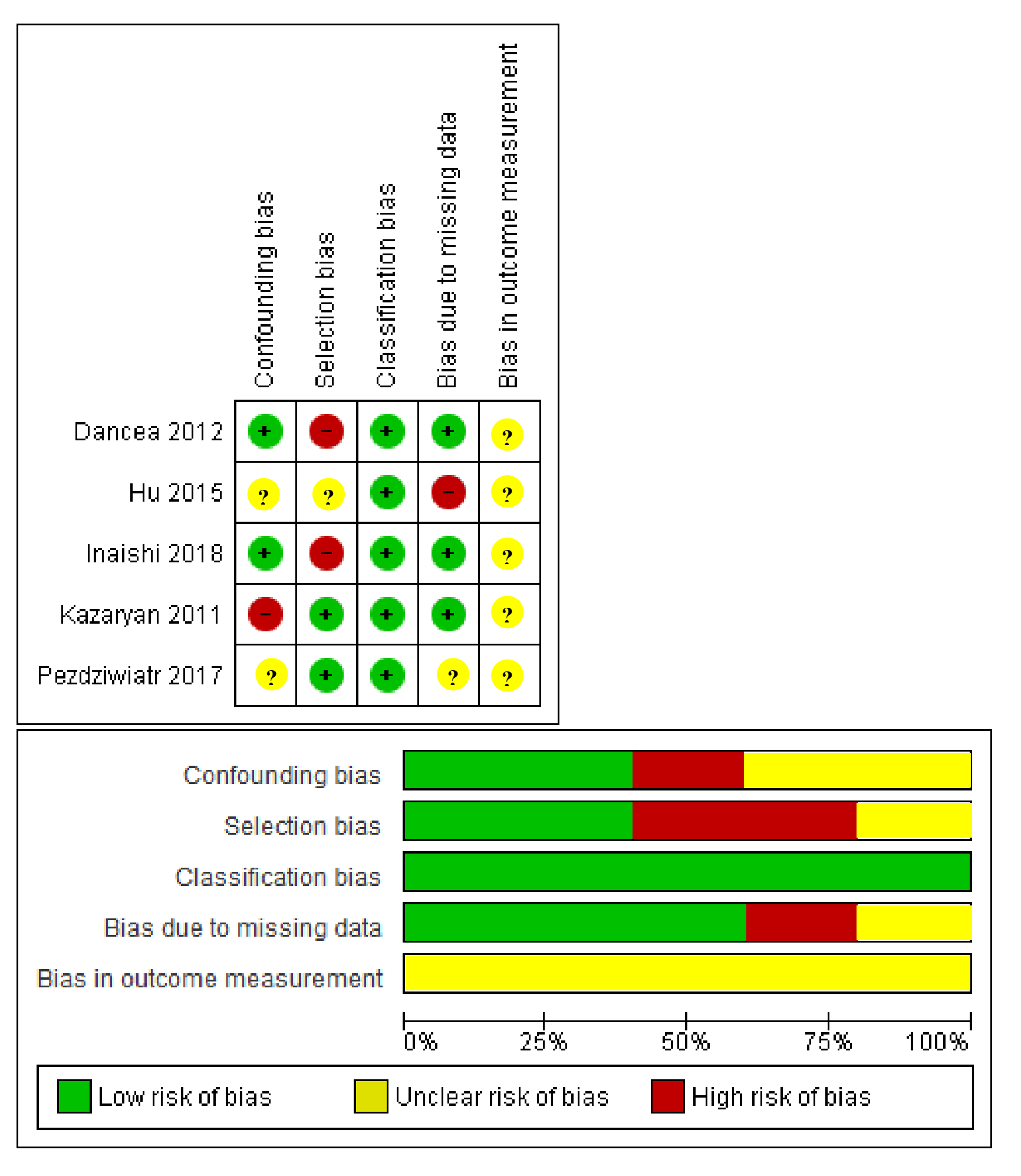


Supplementary Figure 2. Risk of bias in individual studies and summary

Table 1. Summary table of mean intervention time

| Mean time of intervention(min) | Obese | Non-obese | P-value |
| --- | --- | --- | --- |
| Kazaryan | 90(45-167) | 72((32-230) | 0.045* |
| Dancea | 124.5(111-170) | 125(106-143) | 0.45 |
| Hu | 125.12 | 95.31 | <0.001* |
| Inaishi | 107 | 111 | 0.79 |
| Pezdziwiatr | 93.5(34.1) | 88.8 (40.6) | 0.14 |

The values between brackets are SD or interquartile ranges
